# 

**Published:** 1882-02

**Authors:** 


					﻿FEBRUARY, 1882.
TWENTY-NINTH YEAR.
IIAEIJS
JOURNAL
or
WJR AT TH
E. H. GIBBS, A.M., M.D., Editor,
No. 141 EIGHTH STREET (Near Broadway), NEW YORK.
) TERMS: $2 a Year, or $1 for Six Months. Single nnmlier. 20 cts. <
5	C
				

